# Characterisation of the Small RNAs in the Biomedically Important Green-Bottle Blowfly *Lucilia sericata*

**DOI:** 10.1371/journal.pone.0122203

**Published:** 2015-03-24

**Authors:** Cherie Blenkiron, Peter Tsai, Lisa A. Brown, Vernon Tintinger, Kathryn J. Askelund, John A. Windsor, Anthony R. Phillips

**Affiliations:** 1 Department of Molecular Medicine and Pathology, Faculty of Medical and Health Sciences, University of Auckland, Auckland, New Zealand; 2 Department of Surgery, Faculty of Medical and Health Sciences, University of Auckland, Auckland, New Zealand; 3 Bioinformatics Institute, University of Auckland, Auckland, New Zealand; 4 Department of Anthropology, University of Auckland, Auckland, New Zealand; 5 Maurice Wilkins Centre for Biodiscovery, University of Auckland, Auckland, New Zealand; 6 School of Biological Sciences, University of Auckland, Auckland, New Zealand; Kunming University of Science and Technology, CHINA

## Abstract

**Background:**

The green bottle fly maggot, *Lucilia sericata*, is a species with importance in medicine, agriculture and forensics. Improved understanding of this species’ biology is of great potential benefit to many research communities. MicroRNAs (miRNA) are a short non-protein coding regulatory RNA, which directly regulate a host of protein coding genes at the translational level. They have been shown to have developmental and tissue specific distributions where they impact directly on gene regulation. In order to improve understanding of the biology of *L*. *sericata* maggots we have performed small RNA-sequencing of their secretions and tissue at different developmental stages.

**Results:**

We have successfully isolated RNA from the secretions of *L*. *sericata* maggots. Illumina small RNA-sequencing of these secretions and the three tissues (crop, salivary gland, gut) revealed that the most common small RNA fragments were derived from ribosomal RNA and transfer RNAs of both insect and bacterial origins. These RNA fragments were highly specific, with the most common tRNAs, such as GlyGCC, predominantly represented by reads derived from the 5’ end of the mature maggot tRNA. Each library also had a unique profile of miRNAs with a high abundance of miR-10-5p in the maggot secretions and gut and miR-8 in the food storage organ the crop and salivary glands. The pattern of small RNAs in the bioactive maggot secretions suggests they originate from a combination of saliva, foregut and hindgut tissues. Droplet digital RT-PCR validation of the RNA-sequencing data shows that not only are there differences in the tissue profiles for miRNAs and small RNA fragments but that these are also modulated through developmental stages of the insect.

**Conclusions:**

We have identified the small-RNAome of the medicinal maggots *L*. *sericata* and shown that there are distinct subsets of miRNAs expressed in specific tissues that also alter during the development of the insect. Furthermore there are very specific RNA fragments derived from other non-coding RNAs present in tissues and in the secretions. This new knowledge has applicability in diverse research fields including wound healing, agriculture and forensics.

## Introduction

*Lucilia sericata* larvae are commonly known as green-bottle blowfly maggots and are an important species in forensics, agriculture and biomedicine [1,2]. Their ability to assist in wound debridement has been exploited for centuries and they are still used today in the treatment of chronic skin wounds and ulcers to promote healing [3]. *Lucilia* have also proven useful in forensics for estimation of post-mortem intervals [4]. Conversely in agriculture, *Lucilia*, both *L*. *sericata* and to a greater extent *L*. *cuprina*, are parasites of sheep causing blow-fly strike which has detrimental economic effects worldwide [5,6].

Medicinal *L*. *sericata* maggots are believed to have a multifactoral influence on wound healing. Initially believed to be due to the mechanical eating of dead tissue, (debridement), they are now thought to mostly function through their biochemically active excretions and secretions (ES) [7]. The ES has antimicrobial activity [8], protease activity to digest dead wound eschar [9], and even has a direct effect on cells to promote skin wound healing [10]. Studies of *L*. *sericata* ES have focused on the identification of molecules such as amino acids and fatty acids which may play a role in the wound healing [11,12]. Proteins are also involved, for example, a chymotrypsin is reported to degrade dead wound eschar and has the ability to break up bacterial biofilms which are often formed when a wound is infected [13,14]. A nuclease has been identified that can also degrade bacteria biofilms by breaking down their DNA component [15]. The secretions from sibling species *L*. *cuprina* have also been reported to have anti-microbial activity, suggesting that this may be a common feature of fly larvae [16].

The full genome for *L*. *sericata* is not publically available, only the mitochondrial genome is published [17]. Some short DNA sequences have also been released for use in species identification in forensics [1,18]. Due to the importance of this species, the transcriptomes of the developmental stages and dissected salivary glands have recently been published [19]. An expressed sequence tag transcriptome has also been assembled for *L*. *cuprina* [20]. Gene expression analysis of *Lucilia* has already shown great value as it is accurate in developmental stage estimation for use in forensics [21].

The small RNA profiles in multiple flying insects such as *Drosophila* [22], honey bee (*Apis Mellifera*) [23] and mosquito (*Anopheles; Aedes; Culex*) [24,25] have recently added to the better understanding of their development as well as ability to transmit various diseases as vectors. Therefore, to broaden the biological knowledge of *L*. *Sericata* we have performed small RNA-sequencing on their larvae tissues and ES. When the data was matched to known small RNA databases we identified both common and tissue specific RNAs, derived from various families of annotated small RNAs. The abundant small RNAs were then were assayed across developmental stages of *L*. *sericata* and validated in the dissected tissues by droplet digital RT-PCR.

## Materials and Methods

### Lucilia sericata

Eggs and Instar 2/3 larvae were provided for sterilisation and dissection by Consultant Entomologist Dr Dallas Bishop, Upper Hutt, New Zealand. She also provided the developmental stages of the insect, as determined by visual inspection of morphology, snap frozen on dry-ice. The eggs were laid onto liver, removed and sent by overnight courier to our laboratory with an ice-pack to delay hatching. A colony was also established in Auckland, using the same egg supply and rearing techniques, by Mr Vernon Tintinger. All eggs and larvae were maintained at ambient temperature.

### Larvae sterilisation

All equipment and reagents were autoclaved for sterility prior to use. Insect handling was performed in a laminar flow hood and maggots were stored in a sealed box on plates at room temperature.

Eggs were surface sterilised upon arrival approximately 16 hrs after oviposition to mimic the human clinical application state by immersion in 1% sodium hypochlorite for 5min followed by 5min in 70% EtOH with a final rinse in dH_2_O. Eggs were transferred to sterile 10% sheep blood, BHI agar, Colombia blood agar base plates (CBB) (Per L: 7.7g calf brain, 9.8g beef heart, 15g proteose peptone, 2g dextrose, 10g sodium chloride, 2.5g Disodium phosphate, 30g agar, 10g pancreatic digest of casein, 5g yeast extract, 3g beefheart infusion, 1g corn starch, 100ml difibrinated sheep blood). Larvae were hatched in a sealed sterile box. After 48 hours the instar 2 maggots were re-bleached briefly in 0.5% sodium hypocholorite and rinsed in 70% EtOH as for the eggs and plated overnight on soy agar plates (Per L: 15g pancreatic digest of casein, 5g papaic digest of soybean, 5g sodium chloride, 15g Agar) to ensure the blood agar was removed from their systems along with potential food-derived animal RNAs.

At each stage of growth, sample sterilised eggs/maggots were crushed and streaked on CBB plates and grown at 37°C overnight to confirm there was no surface bacterial contamination.

### Larvae dissection and excretion/secretion collection

On day three after eggs bleaching, at instar 2/3, maggots were used for collection of excretions and secretions (ES) and dissections. Prior to use, maggots were again washed in 70% EtOH for 5 min, rinsed in dH_2_O and blotted dry.

**Fig 1:**
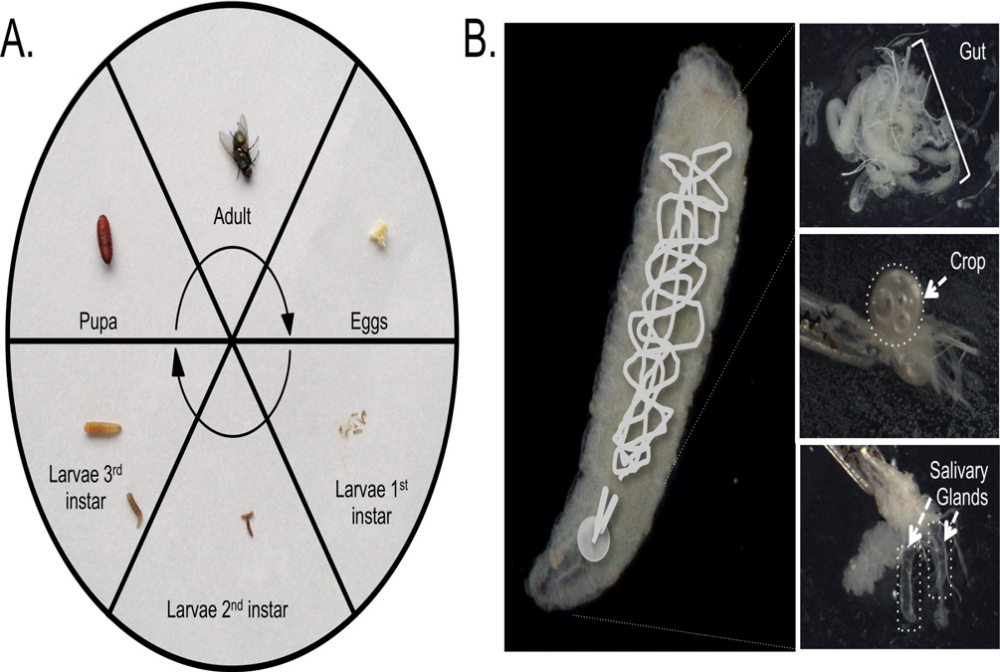
Lifecycle and anatomy of *Lucilia sericata*. A. Lifecycle of the blow-fly from eggs through to adult. B. Anatomy of a maggot showing the location of the gut, crop and salivary glands. The gut was sampled from the whole specimen shown. The crop is the round organ filled with air-bubbles which was further dissected away from the mouthparts, highlighted by the dotted-line circle. The salivary glands are the Y-shaped tubes shown here encircled by dotted lines, still attached to the mouthparts.

To isolate the salivary glands, crop and gut we used a dissecting microscope. Maggots were anaesthetised on ice and quickly placed in a sterile petri dish containing sterile water. Forceps were used at the proximal and distal ends to hold the maggot and pull laterally, ensuring a good hold on the mouthparts. Whole salivary glands, then the diverticulated foregut crop and lastly gut were dissected out and placed into 0.5mL of Trizol LS (Fig. 1).

ES was collected in by placing batches of 50 larvae in 250μL dH_2_O at room temperature in the dark for 1 hour. Maggots were removed and ES spun 5min 2,500g to pellet debris and filtered through a 0.2μM syringe filter. Trizol LS was added at a volume of 3:1 as per manufacturers recommendations.

Representative insects from each of the developmental stages (Fig. 1), as determined by time post-hatching and larval size/morphology by Entomologist Dallas Bishop were snap frozen and then homogenized in 1mL Trizol using a Bullet Blender storm.

### RNA isolation

RNA was extracted from the Trizol samples with a modified Purelink RNA mini kit (Life Technologies) protocol which ensures retention of the small RNA fraction by adding an equal volume of 100% EtOH to the aqueous phase prior to addition onto the filter column. RNA was DNase I treated prior to sequencing using Turbo DNase-free (Ambion). RNA quantity and quality were assessed by Nanodrop and Experion bioanalyser chip (BioRad).

### 2S rRNA depletion

Modified from the method of Seitz *et*. *al*. from *Drosophila* [26], a complementary biotinylated oligo, 5′-biotin-TCA ATG TCG ATA CAA CCC TCA ACC ATA TGT AGT CCA AGC A-3’, was bound to magnetic beads to deplete tissue RNA (SG, Crop, Gut) of the abundant 2S rRNA fragment. This oligo sequence was based on the most common 2S rRNA read identified in a pilot sequencing run and identical to *Drosophila*.

20 pmol oligo synthesised by IDT was coupled to 0.8mg of M279 streptabeads beads (Life Technologies, NZ) washed prior in 0.5xSSC, by mixing on ice for 30 min. Uncoupled oligos were removed by placing the beads on a magnet and washing once with 0.5x SSC, resuspending in a volume of 80μL and then incubating at 65°C for 5 min then quenching on ice.

To the coupled beads we added 5μg total RNA denatured previously at 85°C for 5min, then standing for one hour at 50°C. After depletion the rRNA bound beads were removed using a magnet and the supernatant retained. RNA was extracted from the depleted RNA using an Ambion miRvana column (Life Technologies, NZ) to enrich for the <200bp fraction as per manufacturers instructions.

### RNA sequencing

Sample inputs were 9.2ng enriched depleted SG, 25.4ng depleted gut, 58.0ng depleted crop, 246.8ng whole ES RNA, all collected from sterilised Instar 3 animals. Small RNA libraries were prepared using a TruSeq small RNA sample preparation kit (Illumina) as standard and run on a MiSeq with two samples per lane, 2x25bp paired end reads. Sequencing was performed as a service by New Zealand Genomics Ltd. The data sets supporting the results in this article are available from the Sequence Read Archive, project number SRP028914.

### Sequencing data analysis

MiSeq Reads were linker sequence trimmed, quality trimmed then self-aligned into unique reads. The reads were BLAST against miRBase V19 [27], the Functional RNA database (fRNAdb) [28], and the genomic tRNA database (gtRNAdb) [29] allowing a single mismatch within the query sequence. Unmatched reads from the top ten most common URs by count were BLAST searched against GenBank, nr, and RefSeq databases for all species for identification. Due to the lack of a genome of alignment and the presence of contaminating bacterial RNAs, miRNA abundances were normalised to the total number of reads matching to miRBase V19 content.

### Droplet digital RT-PCR (ddPCR) validation

We used ddPCR to validate the tissue specificity and developmental stage expression of selected miRNAs. Briefly, RNA was isolated from whole samples at seven developmental stages after homogenisation in Trizol with a Purelink Mini kit (Invitrogen) following the protocol for total RNA or from dissected tissues as previously detailed. Developmental stages were RNA extracted in triplicates from individuals (pupae/adult) or pools of insects. The dissected tissues were tested in singleplex due to the lack of excess source material and six replicate ES samples, collected from Instar 1 animals, were assayed. RNA was DNase treated using Zymo DNA-free RNA kit (Zymo Research) and RNA yield was assessed by a Qubit 2.0 fluorometer (Life Technologies) for accuracy.

cDNA was synthesised from 100ng RNA using the Qiagen miScript cDNA kit and diluted 1:10 to 1:4000 with distilled water before use. PCR was performed with custom small RNA primers and predesigned miScript miRNA primer assays (Qiagen) using ddPCR EvaGreen Supermix (Bio-Rad). Reactions were carried out with 4μl of the diluted cDNA in a 20μl final volume, then mixed with 70μl Evagreen droplet generation oil for droplet synthesis using the QX200 system. From this, 40μl of PCR-oil mix was cycled as per manufacturers recommended conditions and read on a QX200 droplet reader. Positive droplet counts as determined by Quantasoft software with samples with less than 10,000 droplets discarded and repeated. The quantification is presented as copies per μl of PCR mixture adjusted for the cDNA dilution factor. The use of ddPCR which does not require a reference gene is ideal for this application due to a lack of genome for *L*. *sericata* to assess conservation of standard small RNAs such as U6.

## Results

Small RNA-sequencing was performed on tissues from instar 3 larvae and also from their bioactive ES (Fig. 1). A pilot sequencing run of an unsterilised *L*. *sericata* salivary gland (SG) library was found to contain a single unique read at a very high abundance where of 2.71M total reads, 91% of these matched to this single RNA. This read aligned to the 30nt 2S rRNA which is reported to be present in all Diptera such as *Drosophila* [30]. In order to remove some of this 2S rRNA sequence from the subsequent tissue RNA samples, we performed a targeted oligo-depletion step prior to library preparations as per protocols used for *Drosophila* [26]. This proved successful and depleted the sequence from accounting for 91% of all counts in SG down to 50%.

Our initial pilot run also included an unsterilised excretion and secretion (ES) library which was found to contain an unexpectedly large amount of bacterial RNA. Medicinal maggots are used in the clinic after a process designed to generate “sterile” larvae, which we undertook to replicate by bleaching of eggs and then bleaching 3-day old larvae before collection of ‘sterilised’ tissue and ES samples. This bleaching is essentially a surface sterilisation process. Four tissue specific libraries were prepared from the instar 2/3 *L*. *sericata* larvae for ES, salivary gland (SG), GUT and CROP (Fig. 1). The latter three tissues were 2S rRNA depleted before sequencing. Each library generated between 4.4–7.6M reads (Table 1). Most reads were 26nt in length after adaptor and quality trimming, although we expect this to be size-biased by the high abundance of the 30nt insect 2S rRNA. The complexity of the libraries in terms of ‘singleton’ reads, that is unique reads (UR) with a single count in the sample, varied across libraries ranging from 68–75% of all UR but accounting for only 3–6% of all counts.

**Table 1:** Summary of counts per library and unique reads once self-aligned.

	Total data set		Singletons		miRBase^a^ matches		gtRNAdb matches^b^		Ribosomal RNA matches		Insect matches		bacteria matches	
Sample	Counts	UR	Counts	UR	Counts	UR	Counts	UR	Counts	UR	Counts	UR	Counts	UR
ES all data	6785399 (100%)	289464 (100%)	197489 (2.9%)^c^	197489 (68.2%)^d^										
ES matched	4462642 (65.8%)	10621 (3.7%)	3731 (0.01%)^e^	3731 (35.1%)^f^	2287 (0.05%)	127 (1.2%)	887957 (19.9%)	5909 (55.6%)	3518995 (78.9%)	3601 (33.9%)	4016110 (90.0%)	4615 (43.5%)	442048 (9.9%)	5430 (51.1%)
SG All data	4439026 (100%)	317864 (100%)	233500 (5.3%)	233500 (73.5%)										
SG matched	3048999 (68.7%)	9558 (3.0%)	3516 (0.1%)	3516 (8.3%)	16146 (0.53%)	423 (4.4%)	560570 (18.4%)	4302 (45.0%)	2451184 (80.4%)	3801 (39.8%)	2678854 (87.9%)	4802 (50.2%)	368505 (12.1%)	4179 (43.7%)
GUT All data	7487282 (100%)	450326 (100%)	328450 (4.4%)	328450 (72.9%)										
GUT matched	2676481 (35.7%)	14737 (3.3%)	7602 (0.2%)	7602 (51.6%)	23071 (0.86%)	409 (2.8%)	1321365 (49.3%)	12729 (86.4%)	1318496 (49.3%)	4117 (27.9%)	2494445 (93.2%)	8318 (56.4%)	172922 (6.5%)	5839 (39.6%)
CROP All data	7563354 (100%)	599206 (100%)	450769 (6.0%)	450769 (75.2%)										
CROP matched	2700838 (35.7%)	14918 (2.5%)	4758 (0.2%)	4758 (31.9%)	11568 (0.43%)	386 (2.6%)	1713100 (63.4%)	9101 (61.0%)	438935 (16.3%)	3785 (25.5%)	497270 (18.4%)	4522 (30.3%)	1722144 (63.8%)	13256 (88.8%)

^a^matched reads that align to known miRNAs in miRBase V19 for any species,

^b^matched reads that align to the genomic transfer RNA database for any species,

^c^percentage relative to total unmatched counts;

^d^percentage relative to total unmatched UR;

^e^percentage relative to total matched counts;

^f^percentage relative to total matched UR.

Reads were BLAST searched against three small RNA databases (miRBase, fRNAdb and gtRNAdb) to identify known annotated small RNAs and their species of origin (Table 1). Only 2.5–3.7% of UR matched to these databases, but these comprised 35–69% of all counts in the samples. Bacterial RNAs comprised 40–89% of all URs in the four libraries with the highest proportion in CROP (88.8%) and surprisingly the lowest in GUT (39.6%). Conversely, the CROP only contained 30.3% of UR and 18.4% of read counts from insect origins.

When we focused on the database matched insect-derived small RNAs, each sample had a slightly different profile of RNA types (Table 2). The most common small RNA by UR was ribosomal RNA (rRNA) in all samples except for the GUT, which had a higher number of transfer RNA (tRNA) URs (55.6%). The single UR with the highest count in all libraries was either the 2S rRNA or tRNA GlyGCC. Indeed the 2S rRNA fragment, even with the depletion step included in the tissue sample processing, still accounted for 74% of matched counts in SG although only 11–14% of GUT and CROP counts.

**Table 2:** Summary of matched reads to different types of non-coding RNA.

	Ribosomal RNA		Transfer RNA		Signal Recognition Particle		miRNA		‘Other RNA' Insect		Transfer Messenger RNA		RNAse P		‘Other RNA' Bacteria		Unknown		Total	
*Sample*	*UR*	*Count*	*UR*	*Count*	*UR*	*Count*	*UR*	*Count*	*UR*	*Count*	*UR*	*Count*	*UR*	*Count*	*UR*	*Count*	*UR*	*Count*	*UR*	*Count*
**ES**																				
All^a^	3601	3518995	6228	919430	55	303	130	2287	96	1194	109	17236	134	538	115	579	140	655	**10608**	**4461217**
Insect	2563	3505802	1751	506339	9	45	130	2287	61	1139	0	0	0	0	0	0	101	498	**4615**	**4016110**
Bacteria	652	10571	4374	412866	46	258	0	0	0	0	109	17236	134	538	115	579	0	0	**5430**	**442048**
**SG**																				
All	3801	2451184	4680	575440	45	179	418	16146	87	354	126	6073	81	313	96	242	255	2646	**9589**	**3052369**
Insect	2832	2443157	1392	218754	8	102	418	16146	64	289	0	0	0	0	0	0	88	136	**4802**	**2678854**
Bacteria	679	5640	3160	356170	37	77	0	0	0	0	126	6073	81	313	96	242	0	0	**4179**	**368515**
**GUT**																				
All	4117	1318496	9201	1321365	81	472	391	23071	197	4636	195	4494	111	248	179	1053	265	2646	**14737**	**2676481**
Insect	3020	1293594	4630	1172835	41	291	391	23071	172	4503	0	0	0	0	0	0	64	151	**8318**	**2494445**
Bacteria	822	18721	4494	148227	37	178	0	0	0	0	195	4494	111	248	179	1053	1	1	**5839**	**172922**
**CROP**																				
All	3785	438935	13061	1713100	96	429	371	11568	56	222	271	54582	226	1944	270	2994	173	2000	**18309**	**2225774**
Insect	2607	404716	1483	80783	2	23	371	11568	29	149	0	0	0	0	0	0	30	31	**4522**	**497270**
Bacteria	1005	31540	11390	1630678	93	405	0	0	0	0	271	54582	226	1944	270	2994	1	1	**13256**	**1722144**

^a^Includes insect, bacterial, other and unknown species matches.

The top ten most common URs in each of the four samples were further BLAST searched against RefSeq databases (S1 Table). Firstly, this confirmed that much of the bacterial RNA in the ES was successfully removed by our surface sterilisation of the maggots. This was evidenced by the reduction from all ten reads matching to bacteria in the pilot unsterilised ES down to only three of ten reads post-sterilisation. In the tissue libraries, SG, GUT and CROP, we see variation in the number of bacterial and insect matches. All GUT top ten reads were insect-derived, whereas the CROP sample was almost the direct opposite with 9 of the 10 matching bacterial RNAs. In both ES and CROP we identified a 23S rRNA fragment which maps uniquely to the bacterium *Proteus mirabilis*. Another 23S rRNA fragment derived from closely related genus *Providencia* was also detected at high abundance in the SG and CROP samples.

**Fig 2:**
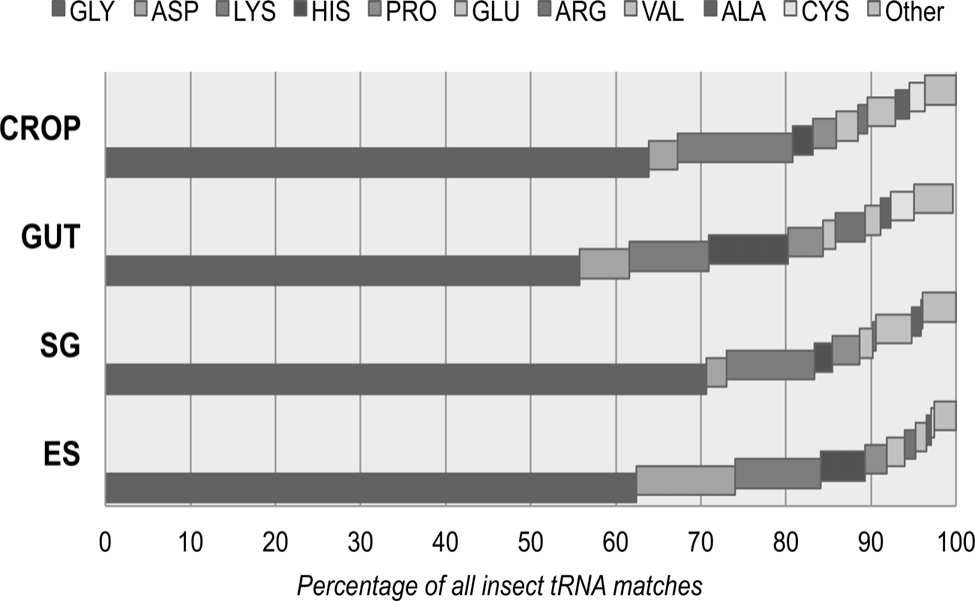
Summary of insect tRNA counts in *L*. *sericata* tissue libraries. The proportion of each tRNA in the different tissue libraries as normalised to the total number of insect tRNA matches in that sample. tRNAs are denoted by their amino acid name.

We next looked at the patterns of insect tRNA fragments that were found in each sample (Fig. 2; S2 Table). A tRNA GlyGCC was the most common fragment identified, accounting for 55–70% of all insect tRNA counts. Other common tRNAs were AspGTC, LysCTT, LysTTT, HisGTG, ProCGG and ValCAC. Interestingly when the fragments are matched to the known *Drosophila* mature tRNA sequences they are predominantly at the 5’ end, particularly when the tRNA is highly abundant. The fragmentation patterns for tRNAs were generally consistent across samples with the only differences seen for those with lower read counts suggesting that they are random degradation products.

**Fig 3:**
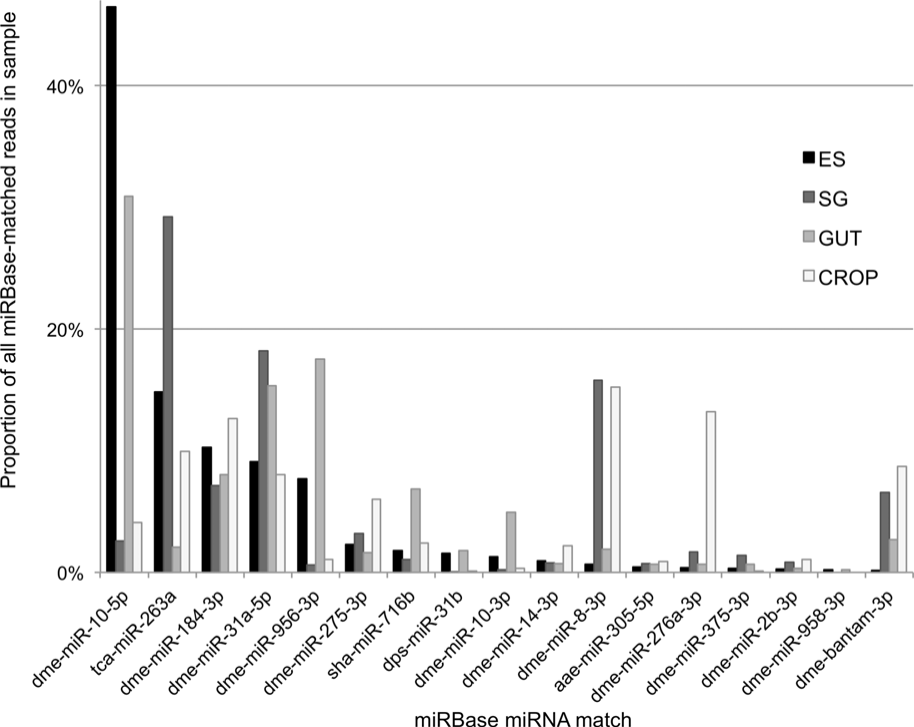
Abundance of miRNA counts in *L*. *sericata* tissue libraries. Matching of the counts to miRBase V19 identified known annotated miRNAs from insect and mammals. The counts per matched miRNA as a proportion of all counts that matched to miRBase are shown in the four tissues. Species nomenclature is used for the miRNA with the perfect matches to the *L*. *sericata* reads: dme, Drosophila melanogaster (fruit fly); tca, Tribolium castaneum (red flour beetle); sha, Sarcophilus harrisii (Tasmanian Devil); dps, Drosophila pseudoobscura (fruit fly); aae, Aedes aegypti (Mosquito).

Finally we focused on the identification of known miRNA homologues by BLAST searching against miRBase V19 (Fig. 3; S3 Table). The miRNA matches for each sample comprised only a small proportion (1.2–4.4%) of the total matched URs. This low number is due to the high levels of contaminating bacterial RNAs and insect rRNA/tRNAs which dominated the samples. The miRNA profiles between samples varied distinctly. The most common miRNA in the ES library for example was miR-10-5p which accounted for 46% of all matched miRNA counts and was also the highest in GUT (31%). However, this only contributed 2.6% in SG and 4% in CROP. This variation continued with the second most common in the ES, miR-263a, comprising 15% of all miRNA ES reads. This was the most abundant miRNA in SG at 29% but only accounted for 2% in GUT and 10% in CROP. Looking at the solid tissues, rather than the secreted ES sample, we see many highly abundant *Drosophila* miRNAs were also found in *L*. *sericata* larval tissues, such as miR-8, miR-31a, bantam and miR-275 [22]. In our data we also found a small number of reads which matched to miRNAs in miRBase but appear to be misannotated small RNA fragments. For example, sha-miR-716b from *Sarcophilus harrisii* (Tasmanian devil) also matched to fRNAdb as a fragment of insect large subunit rRNA. To note, we have also identified some low read count mammalian miRNAs in our tissue libraries, such as miR-486-5p, a red blood cell abundant miRNA, that we hypothesise to be derived from the sheep blood agar which was provided as a food source for the maggots. Unfortunately, due to the current lack of a genome for *L*. *sericata* and the limited depth of our RNA-seq data we are unable to identify novel miRNAs in the species.

**Fig 4:**
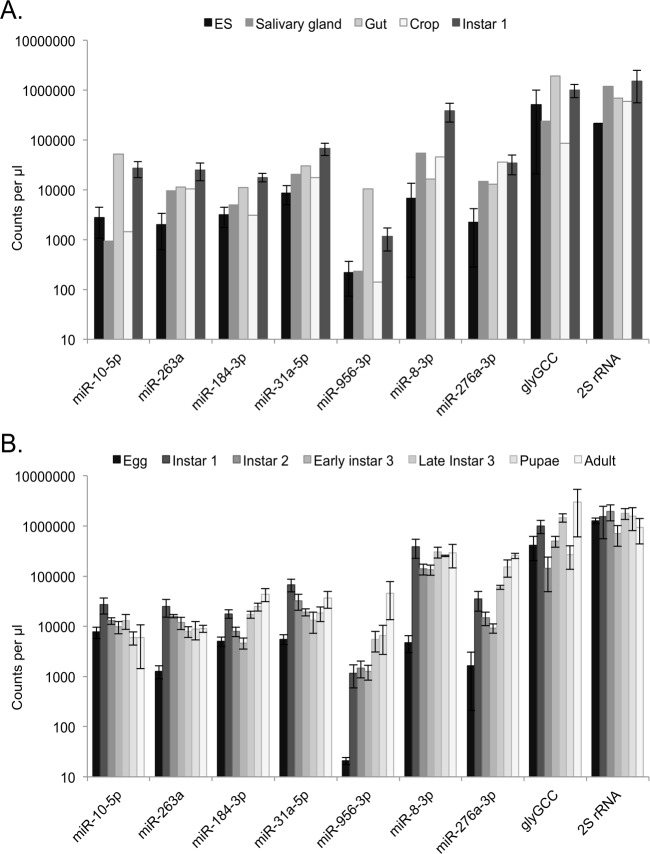
Droplet digital RT-PCR validation of small RNAs in tissue samples and through *L*. *sericata* developmental stages. A. Assessment of small RNA abundances in dissected tissues, instar 1 ES and whole instar 1 larvae and B. throughout developmental stages. Average of replicates (for ES and developmental stages) plotted and error bars are standard deviations.

In order to validate and expand on the findings from the RNA-seq we performed droplet digital RT-PCR on RNAs from dissected tissues and also from various developmental stages of *L*. *sericata* from whole eggs through larval stages to pupae and eventually to newly emerged adult fly (specifically ES, SG, Gut, Crop, eggs, instar 1, instar 2, early instar 3, late instar 3, pupae, and newly emerged Adult fly). We assessed the highly common 2S rRNA fragment, tRNA GlyGCC 5’ fragment, and 7 miRNAs in these samples (miR-10-5p, miR-184-3p, miR-31a-5p, miR-263a, miR-8-3p, miR-276a-3p, miR-956-3p) (Fig. 4). This method was chosen to negate issues with finding a suitable small RNA normaliser for use in standard quantitative RT-PCR due to the lack of genome for *L*. *sericata*. The ddPCR analysis validated the presence of the identified miRNAs and RNA small fragments in the dissected tissues. It also highlighted the variability seen for small RNAs that are commonly used as qRT-PCR reference genes, such as 2S rRNA, between tissue types. Overall, in agreement with the sequencing data, the miRNAs are less abundant in ES than rRNA or tRNA suggesting that the bulk of the insect derived RNA found in ES comprises these small RNA families.

We also detected differences in many of the small RNAs assayed through developmental stages. For the most part, their abundance is lowest in eggs with an increase after hatching into instar 1 larvae. The miRNAs have specific patterns of expression throughout the developmental process with miR-10-5p gradually declining in abundance and miR-956-3p having the opposite pattern of expression.

## Discussion

The aim of this study was to use small RNA-sequencing technology to identify the profiles in a range of medicinal maggot tissue samples and in its ES. The standard for analysis of such data is to initially map the reads to the genome of interest before further analysis, discarding any which are unmapped. In our case this was not possible due to lack of a complete published genome. We note therefore that this dataset is limited to common subcategories of small RNAs from orthologous insects. However because of our non-genome-mapping approach to the analysis we were able to identify small RNAs in an unbiased manner, including both fly and bacterial sequences. The RNA component present in ES appears to be derived from all its potential tissue sources (gut, saliva and crop regurgitation). In terms of the overall RNA profiles, tRNA and miRNA patterns, the SG and CROP source libraries are most similar to one another, possible due to the close proximity of these tissues in the maggot foregut.

### Bacterial small RNA signatures

Small RNA-seq of *L*. *sericata* tissues and ES highlighted the presence of a varied profile of commonly annotated small RNAs, derived from insect, bacterial and food sources. The surface bleaching of eggs and larvae into medicinal-grade maggots, did as expected and removed a large proportion of bacterial burden; however the database read matches still identified bacterial RNAs in the tissues and ES. The bacterial content was highest in the crop, a food storage organ [31], which had almost 10-fold more bacterial RNA reads than the gut sample. The crop is where pathogenic bacteria are killed so that the gut remains for the most part, sterile [32]. Thus the crop acts as a major reservoir for pathogens and bacteria. Read matches from the CROP library pinpointed the presence of *Proteus mirabilis* 23s rRNA, a known symbiotic bacterium in flies [33] and also *Providencia*, identified as an insect pathogen in *Drosophila* [34]. The bacterial rRNAs and tRNAs in all of the libraries show very specific fragmentation patterns. The origins of these RNAs could be due to either bacterial autolysis [35] or even due to specific RNA secretion from the live bacteria as recently reported for *Mycobacterium tuberculosis* [36].

### Maggot small RNA profile

Here we have shown for the first time that the bioactive maggot ES contains RNA. Furthermore the RNA profiling of the three tissues from the gastrointestinal tract of *L*. *Sericata* has shown that each has a distinct pattern of small RNA expression. The most common reads in all samples were fragments from 2S rRNA and tRNA GlyGCC. The 2S rRNA is found only in Diptera, processed as a 30nt fragment from the 5S rRNA [37]. It is commonly used as a loading control for miRNA/siRNA expression analysis as it is ubiquitously present in all tissues at a high level [38,39], its high abundance causing a skew in overall size profiles in the sequencing libraries. This 2S rRNA was present at high levels in all *L*. *sericata* samples we assayed by RNA-seq and across the developmental stages of the insect by ddPCR. Its function, other than as a ribosomal component is unknown. The ddPCR validation showed that the 2S rRNA was present in all tissues and the ES, at levels 13 to 22-fold higher than the most abundant miRNA assayed.

The second most common single read in the RNA-seq libraries was a very specific fragment (tRF) from the 5’ end of the GlyGCC tRNA. This tRF was in particular highly abundant in the GUT and ES libraries and ddPCR showed it was 37 to 60-fold more abundant in these than any of the other miRNAs assayed. Other 5’ tRFs that had high counts in all tissue libraries were AspGTC, LysCTT and HisGTG. Indeed, all of the tRFs with high count numbers were derived from the 5’ end of the mature tRNAs. Furthermore the dominant fragment for each tRNA was found to be consistent across the different tissues. tRFs have been explored in response to stress in mammals where they can function as novel global translation inhibitors [40,41]. In *Drosophila*, tRFs have been found complexed with the piRNA-binding protein PIWI and have a role in RNA silencing [42]. In the archeon *Haloferax volcanii* these tRFs bind to the ribosome to interfere with peptidyl transferase activity and reduce translation [43]. In the ES we also identified a high abundance of tRFs. This parallels what was seen in mouse serum samples where 5’ tRNA halves are present in 100-300KDa protein complexes, with the most common read being GlyGCC [44]. These protein complexes may therefore provide protection from degradation in biofluids such as serum and ES. Interestingly GlyGCC 5’ tRF is also induced in mammalian endothelial cells after bacterial infection [45]. It was predicted to directly regulate mRNAs, using the same mechanisms as miRNAs, with targets including genes important in the infection response; endothelial barrier, inflammatory response and autophagy [45]. It is therefore possible that secreted tRNA has an anti-bacterial function, perhaps even controlling symbiotic relationships with bacteria such as *Proteus* and *Providencia*. Other fragments identified in addition to these 5’ tRNA fragments were at very low counts and were likely to be non-specific degradation products.

### Maggot microRNA profile

MicroRNA profiling in insects has been performed on many different species from the model organism *Drosophila* to mosquitos and ticks, which are commonly responsible for the transmission of disease in mammals [22,25,46]. Many of these studies have profiled the miRNAs throughout the developmental stages of the insect and/or in dissected tissues. Our RNA-seq data identified 125 known miRNAs in various tissue libraries synthesised from sterilised *L*. *sericata*. These sequence matches were predominantly to miRNAs from flying insects such as *Drosophila* (82; 66%), suggesting that they’re directly derived from the maggot rather than from the bacterial and food source contamination. Some low count mammalian miRNAs were found but these were rare as we ensured that the maggots were starved overnight of their sterile blood food source before sampling.

Each of the tissue libraries had a different profile of miRNAs which in the most part was validated by ddPCR. For example, two anti-apopototic miRNAs in *Drosophila* miR-263a and miR-8 [47] were the most common miRNA reads in salivary glands, and crop respectively in the sequencing data. In line with the finding for Gly GCC being enriched in the ES and GUT samples, these two libraries show similar patterns of miRNA abundances such as miR-10-5p and miR-956-3p.

When the *L*. *sericata* miRNAs are compared to published insect profiles, we see many overlap in terms of their overall abundance or their tissue expression profiles. For example miR-8, miR-184, and bantam are found in all *Drosophila* tissues [22] and miR-184, bantam and miR-263a are present in salivary glands from the tick *Haemaphysalis longicornis* [48].

All of the miRNAs assessed by ddPCR were expressed at relatively low levels in *L*. *sericata* eggs and then rapidly increased in instar 1 larvae (3.5 to 55-fold increase). It appears therefore that miRNAs are switched on during larval development and are likely important in the regulation of this process. Furthermore, most miRNAs slightly declined in levels between instar 1 and early instar 3 before either continuing this decline (e.g. miR-10-5p) or increasing again from late instar 3 to adults (miR-184-3p, miR-31a-5p, miR-276a-3p). This increase in expression at late instar 3 could be associated with feeding and rapid growth. The increase in later larval stages, pupae and adults of these miRNAs was also reported for the silkworm moth *Bombyx mori* [49]. Conversely, miR-10-5p appears to peak at instar 1 and then gradually decline in levels in late instar 3 to adult, which is not recapitulated in the silkworm moth. miR-956-3p, which is expressed higher in the gut and ES compared to the crop and SG, was found at much higher levels in the newly emerged adult fly than in the other developmental stages. This miRNA has predicted gene targets involved in muscle development, growth and signaling in *Drosophila* [50].

The variation in miRNA abundance between the developmental stages proposes the potential for them to be used in aging of maggots. Gene expression analysis has proven valuable in forensics for accurate determination of developmental stages. A set of miRNAs, such as miR-10-5p which decreases through instars and miR-956-3p which increases could therefore be useful in forensic staging. Furthermore miRNAs are more stable than messenger RNAs which are currently assayed [51]. In addition, we have identified various miRNAs, such as miR-8-3p, that are present in all larval stages of *L*. *sericata*. It is possible that novel siRNA-based therapeutics, aimed at the interference of these insect-specific miRNAs might be useful in the treatment of blow-fly infections in agriculture to hinder larval development.

The multimodal effects seen when maggot ES is used in the clinical setting is probably due to their diverse molecular composition, with proteins, fatty acids and peptides all found to be functional in promoting wound healing [8,12–15,52]. The RNA component of ES that we have identified here may also have activity in wound healing. Indeed Wang *et al* discussed the potential for miRNAs to act as antimicrobials by downregulating bacterial gene expression [53]. In the literature, RNA within bioactive insect secretions are limited to the study of the Honey Bee (*Apis*) which produces both honey and royal jelly. The components of the royal jelly secretions have epigenetic effects on gene expression such that a worker bee fed on it will develop into a queen bee. Like the maggot ES, royal jelly has recently been shown to contain specific miRNAs, including miR-184, -276a, -10, -8, -31a and bantam [54], all of which overlap with our maggot ES findings. Furthermore, royal jelly itself is a proposed natural therapy for treatment of chronic wounds [55] and has potent anti-bacterial properties [56].

## Conclusions

The small RNA profile and in particular the miRNA profile in *L*. *sericata* may hold great interest for many research fields from forensics to agriculture. We have identified common subcategories of small RNA in the tissues of *L*. *sericata* and shown that these also vary by developmental stage. Furthermore, these tissues commonly contain specific fragments of ribosomal and transfer RNAs which appear to be more than simply degradation products. Finally, we have shown for the first time that the secretions of *L*. *sericata* maggots contain RNA, which we propose may be directly involved in its potency as an antimicrobial and wound healing agent.

## Supporting Information

S1 Table Top Ten unique reads in maggot libraries matched to RefSeq/GenBank. (XLSX)

S2 Table Summary of tRNA fragment counts from insect-matched reads. (XLSX)

S3 Table Matches of unique reads to miRBase V19 for each tissue library. (XLSX)
